# Automated Sample Preparation and Data Collection Workflow for High-Throughput In Vitro Metabolomics

**DOI:** 10.3390/metabo12010052

**Published:** 2022-01-08

**Authors:** Julia M. Malinowska, Taina Palosaari, Jukka Sund, Donatella Carpi, Gavin R. Lloyd, Ralf J. M. Weber, Maurice Whelan, Mark R. Viant

**Affiliations:** 1 School of Biosciences, University of Birmingham, Birmingham B15 2TT, UK; R.J.Weber@bham.ac.uk; 2 Joint Research Centre (JRC), European Commission, 21027 Ispra, Italy; Taina.PALOSAARI@ec.europa.eu (T.P.); Jukka.SUND@ec.europa.eu (J.S.); Donatella.CARPI@ec.europa.eu (D.C.); Maurice.WHELAN@ec.europa.eu (M.W.); 3 Phenome Centre Birmingham, University of Birmingham, Birmingham B15 2TT, UK; G.R.Lloyd@bham.ac.uk

**Keywords:** automation, sample preparation, in vitro metabolomics, direct infusion mass spectrometry, high-throughput screening

## Abstract

Regulatory bodies have started to recognise the value of in vitro screening and metabolomics as two types of new approach methodologies (NAMs) for chemical risk assessments, yet few high-throughput in vitro toxicometabolomics studies have been reported. A significant challenge is to implement automated sample preparation of the low biomass samples typically used for in vitro screening. Building on previous work, we have developed, characterised and demonstrated an automated sample preparation and analysis workflow for in vitro metabolomics of HepaRG cells in 96-well microplates using a Biomek i7 Hybrid Workstation (Beckman Coulter) and Orbitrap Elite (Thermo Scientific) high-resolution nanoelectrospray direct infusion mass spectrometry (nESI-DIMS), across polar metabolites and lipids. The experimental conditions evaluated included the day of metabolite extraction, order of extraction of samples in 96-well microplates, position of the 96-well microplate on the instrument’s deck and well location within a microplate. By using the median relative standard deviation (mRSD (%)) of spectral features, we have demonstrated good repeatability of the workflow (final mRSD < 30%) with a low percentage of features outside the threshold applied for statistical analysis. To improve the quality of the automated workflow further, small method modifications were made and then applied to a large cohort study (4860 sample infusions across three nESI-DIMS assays), which confirmed very high repeatability of the whole workflow from cell culturing to metabolite measurements, whilst providing a significant improvement in sample throughput. It is envisioned that the automated in vitro metabolomics workflow will help to advance the application of metabolomics (as a part of NAMs) in chemical safety, primarily as an approach for high throughput screening and prioritisation.

## 1. Introduction

New approach methodologies (NAMs) are understood as alternative methods to vertebrate animal testing that provide greater robustness, throughput and/or mechanistic knowledge into risk assessment, thereby enabling more relevant decision making for human health and the environment. Some examples of NAMs include in vitro, in silico, and in chemico approaches, high-throughput screening (HTS), omics technologies or combinations thereof [1,2,3]. In particular, omics technologies are capable of providing comprehensive information on the molecular response of a biological system to external stimuli such as exposure to a chemical. Academia, industry and regulatory bodies are increasingly recognising the benefits of using omics approaches, e.g., for mode-of-action determination, determining potency in the form of points-of-departure and deriving a mechanistic basis to cross-species extrapolation [4,5,6,7,8].

Metabolomics provides the most downstream molecular measurements of the omics technologies, enabling a phenotypic readout of a biological system at a particular time [9]. Consequently, metabolomics is more closely related to current approaches in toxicity testing that measure final manifestations of toxicity through adverse or “apical” endpoints (e.g., changes in body weight). There have been several proof-of-principle studies employing metabolomics to regulatory-relevant scenarios such as read-across [6,10]. Similar to high-throughput transcriptomics (HTTr), applying metabolomics in combination with HTS (i.e., large-scale assays employing automation) could provide a tool for rapid identification and characterisation of responses of biological systems to chemical exposure [11], yet would require increased throughput of metabolite extractions.

Developments in solid-phase (e.g., RapidFire by Agilent, Santa Clara, CA, USA) and electro-driven extractions offer increased throughput, however, these approaches are biased towards specific classes of metabolites [12]. Most recently, Meister et al. [13] employed an automated liquid-handling platform (Agilent) for the normalisation of specific gravity of urine samples and extraction of urinary metabolites. Other advances in high-throughput metabolomics include acoustic mist ionisation mass spectrometry, achieving a throughput of < 10 s per sample analysis [14], although at the cost of lower detection sensitivity, higher technical variance and lower confidence in metabolite annotation [15,16,17]. Other high-throughput methods for metabolite data collection include flow injection mass spectrometry (FI-MS) capable of collecting high-quality data in less than a minute [18,19,20]. This technique, however, uses flow rates in the range of microlitres-per-minute as opposed to nESI-DIMS (using nanolitres-per-minute) which affects the desolvation rate during the electrospray process [16,17,21,22,23]. Nonetheless, current nESI-DIMS approaches still require time-consuming sample preparation despite its high-throughput nature; Southam et al. [16] recommended preparation of no more than 20 samples in a batch for nESI-DIMS analysis when manually handling the samples. More recently, Xu et al. [24] coupled capillary microsampling with nESI-DIMS for lipid analysis of only 20 mammalian cells offering a compromise between high analytical sensitivity and high-throughput data acquisition. Nonetheless, capillary microsampling can be time-consuming and requires training to accurately and rapidly (<1 min) sample the cells. Despite this progress, there remains the need for robust high-throughput automated workflows that are both compatible with 96- and/or 384-well microplates and could be employed routinely in HTS.

The objective of this work was to develop an automated workflow for in vitro metabolomics (from only 50,000 hepatocytes of HepaRG cultured in 96-well microplates through to data collection), benefitting from a recently modified nESI-DIMS method for low biomass samples [25]. In this study, we employed an automated laboratory workstation, Biomek i7 (Beckman Coulter), customised to meet the requirements of in vitro metabolomics studies, such as maintaining low sample temperatures (−15 °C) during metabolite extraction to minimise unwanted enzymatic activity. The study characterised the performance of this workflow—in terms of sensitivity and repeatability—under multiple experimental conditions, applying extensive statistical analyses to ensure high quality data were achieved across various scenarios of metabolite extractions. The workflow was subsequently improved and applied to a large cohort HTS experiment in the HepaRG cell line. The workflow presented in this study offers an automated approach for in vitro metabolomics for application in NAMs to advance the pace of chemical risk assessment.

## 2. Results

### 2.1. Evaluation of Sensitivity and Repeatability of the Automated Platform for Intracellular Metabolite Extraction and Analysis

**Figure 1:**
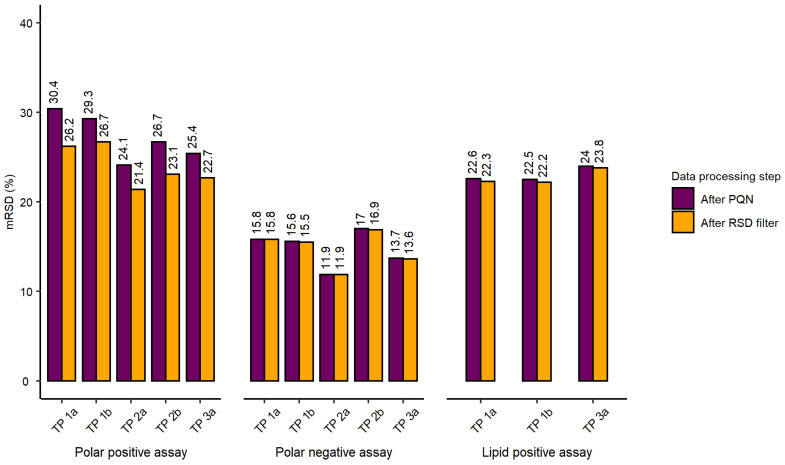
Results presented for all three nESI-DIMS assays (polar positive, polar negative and lipid positive) with 96-well microplates, labelled as “test plates” or “TP”, indicating the order of their extraction (1–3) and position on the instrument’s deck (a-b) following normalisation (termed “After PQN”), and filtering of variable features (termed “After RSD filter”). For each 96-well microplate, median relative standard deviation (mRSD (%)) of spectral feature intensities was calculated before and after the RSD filtering (i.e., removal) of spectral features for which the feature RSDs exceeded 30% in intrastudy QC samples.

The initial evaluation of the workflow included the assessment of total spectral feature counts (after blank subtraction and feature quality filtering), mRSD (%) of spectral feature intensities across the whole dataset and RSD (%) of a feature intensity putatively assigned to the isotopically labelled internal standard across three nESI-DIMS assays (L-tryptophan-d_5_ for polar assay, both ionisation modes, and dodecylphosphorylcholine-d_38_ for lipid positive assay), as shown in Table 1. The analytical sensitivity across three nESI-DIMS assays was high, with total spectral feature counts exceeding 3000 for each assay (immediately after the probabilistic quotient normalization (PQN) step of data processing described in Section 4.1.4). Here, we also included total spectral feature counts after removing technically variable features, i.e., those with RSDs exceeding 30% in intrastudy quality control samples (QCs), termed here “RSD filtered” (Table 1). The feature counts of spectral features retained after RSD filtering were also high, indicating both high analytical sensitivity and repeatability of the workflow across all assays, with few peaks being removed by this particular filter. The repeatability was further characterised by using mRSD (%) of spectral features measured in intrastudy QCs (measuring analytical variation) and biological control samples (measuring the sum of analytical and biological variation), with the latter corresponding to individual wells of 96-well microplates. This metric was proposed by Parsons et al. [26] to evaluate the quality of metabolomics data. The analytical repeatability measured using intrastudy QCs was excellent for both polar negative and lipid positive nESI-DIMS assays, achieving mRSDs of spectral feature intensities below 15% for datasets after PQN and RSD filtering. For the polar positive nESI-DIMS assay, this value was slightly higher than anticipated for the dataset after PQN step (20.9%) which decreased to 17.3% after RSD filtering. This assessment included the variation originating from re-aliquoting of the pooled sample to individual wells of a 96-well microplate, drying, resuspension of intrastudy QCs and subsequent nESI-DIMS analysis. The evaluation of biological control samples was conducted to assess the repeatability of the entire workflow from cell culturing through sampling, metabolite extraction, resuspension of samples and data collection. The repeatability was deemed satisfactory for polar negative and lipid positive nESI-DIMS assays after PQN and RSD filtering (not exceeding our quality threshold of 30% for biological control samples), whilst polar positive nESI-DIMS assay yielded slightly higher variation than expected after PQN (mRSD 31.3%) but decreased after removing more variable features (mRSD 27.6%). Each 96-well microplate in the study was individually assessed with respect to mRSD (%) of spectral feature intensities before and after applying RSD filtering across three nESI-DIMS assays. Figure 1 demonstrates that each microplate (except for TP 1a in the polar positive assay after PQN) met the threshold of 30% for mRSD of spectral feature intensities. For polar negative and lipid positive assays, the removal of more variable features had little effect on the final mRSD (%) of the whole dataset, whilst for the polar positive assay it did result in a notable improvement of the repeatability. The differences between mRSD (%) of tested microplates in each assay were small and most pronounced in the polar positive assay, with the mRSD ranging from 21.4% to 26.7% after RSD filtering.

To further characterise the repeatability of the proposed workflow, RSDs of a spectral feature putatively assigned to each internal standard were calculated: L-tryptophan-d_5_ or dodecylphosphorylcholine-d_38_ as shown in Table 2. The primary purposes of each internal standard were to help identify (and reject) outlying samples that exceed set intensity thresholds and then to assess the final intensity variation of this spectral feature across the whole dataset. The thresholds were calculated using median and median absolute deviation (MAD) of the intensity of that spectral feature (see Section 4.1.4, Equations (1) and (2)). For intrastudy QCs and biological control samples, the RSD (%) values were satisfactory for all three assays, although the polar positive nESI-DIMS assay revealed the highest variation for this feature. This observation is in agreement with the results above, for which the polar positive assay performed most poorly across the three nESI-DIMS assays tested in terms of mRSD (%) of all spectral features.

### 2.2. Evaluation of Experimental Conditions and Intra/Inter-Day Variation of the Automated In Vitro Metabolomics Workflow

Further assessments of the quality of the workflow for potential deployment in HTS included statistical analysis of the following experimental conditions (as shown in Section 4).

- Inter-day repeatability of metabolite extractions from HepaRG samples in 96-well microplates (Test plate (TP) 1a vs. 3a)
- Order of metabolite extraction from 96-well microplates (TP 1a vs. 2a, TP 1b vs. 2b), evaluated within one day, and only relevant to polar metabolites
- Position of 96-well microplate on the sample preparation platform’s deck (TP 1a vs. 1b, TP 2a vs. 2b)

**Figure 2:**
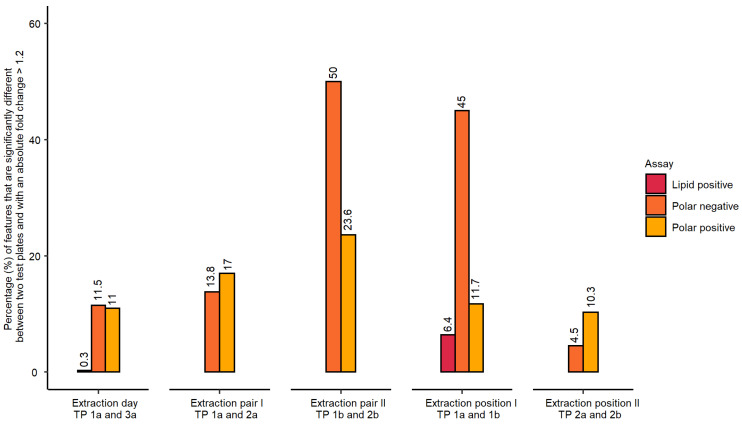
Percentage of metabolic features which were found “not repeatable” across studied experimental conditions measured for polar metabolites (both ionisation modes) and lipids (positive ionisation mode only). The order of extraction of 96-well microplates contributed most significantly to observed differences in feature intensities between the microplates, which triggered a modification of the proposed workflow.

The statistical analysis employed one-way analysis of variance (ANOVA) followed by post-hoc testing with the alpha threshold set to 0.05. The analysis also included fold change calculations between the experimental groups. A metabolic feature was considered outside the permissible threshold (i.e., regarded as “not repeatable”) if the false discovery rate (FDR) adjusted *p*-value was at or below 0.05 and its absolute fold change was above 1.2 (Figure 2). The purpose of including this fold change criterion (common in transcriptomics data analysis) was to reduce the likelihood of falsely assigning a feature as “not repeatable” given the high metabolic similarity of the control samples.

The evaluation of inter-day differences in metabolite extraction revealed a very low percentage of features that were “not repeatable” (Figure 2), i.e., outside the applied thresholds, particularly for lipidomics data (0.3% of 3788 spectral features). However, the percentages of “not repeatable” features were higher than expected for the polar nESI-DIMS datasets (11.5% and 11.0% for negative and positive ionisation modes, respectively, Figure 2). It is worth noting that these differences could have originated from cell culturing, the implemented metabolomics workflow, or a combination of both. For the polar data, an additional parameter in the experimental design was assessed—the order of metabolite extraction from 96-well microplates was prepared on the same day. In this case, the samples in the first pair of 96-well microplates waited 40 min at room temperature to be dried, so that all study 96-well microplates could be dried at the same time. A high percentage of “not repeatable” metabolic features was observed for the second extraction pair of 96-well microplates in the polar negative assay (50.0% of 4781 features for the pair TP 1b and 2b, Figure 2). Similarly, the polar positive dataset revealed a high percentage of metabolic features exceeding our applied threshold (23.6% of 2329 features). Based on these results, this step of the workflow was subsequently modified to reduce the percentage of “not repeatable” metabolite intensities, which is discussed in the next section. The position of a 96-well microplate on the sample preparation platform’s deck revealed low percentages of metabolic features outside the applied thresholds for polar positive and lipid positive assays—11.7%, and 6.4%, respectively. However, for polar negative assay, there was a high number of “not repeatable” features for extraction position I (45%), expected to be a result of the additional waiting period these extracts were subjected to (40 min at room temperature followed by 1 h 15 min at 4 °C). This delay was caused by limitations in available equipment at the time (a single SpeedVac was available with a 6-microplate rotor) (Figure 2). The second pair only included the measurement of polar metabolites and was slightly higher than expected for polar positive assay (10.3%). Next, the study also investigated if there were significant differences between wells located in the edge or centre of each 96-well microplate (Table S1). These differences could in principle result from cell media evaporating more rapidly during the cell culturing and/or the automated liquid handling (i.e., pipetting) occurring imprecisely in the wells located at the edges.

For this reason, each 96-well microplate across all 3 nESI-DIMS assays was assessed using Welch’s *t*-test (*p*-value threshold 0.05 with FDR correction) and calculations of fold changes. No features were found outside of the applied thresholds for the lipid positive assay (i.e., there were no “not repeatable” features), whilst for the polar negative assay only 2 out of 4781 features were found “not repeatable” (TP 2a, Table S1). For the polar positive assay, 96-well microplates TP 2a and 2b revealed that 7.2% and 0.6% of features differed in intensity between edge and centre wells, respectively, whilst the remaining 96-well microplates did not reveal any such differences. The results suggest that cell culturing and liquid handling do not contribute to the observation of significantly differing feature intensities between sample wells located at the edge or centre of a microplate (producing only a few “not repeatable” features), hence all 96 wells could be used for the subsequent cohort study.

### 2.3. Demonstration of the Developed Workflow for High-Throughput Metabolomics Studies

The characterisation of the implemented workflow revealed a high percentage of “not repeatable” features (i.e., features with adjusted *p*-values at or below 0.05 and absolute fold changes > 1.2) when evaluating the order of 96-well microplates being extracted. Therefore, this step of the workflow was optimised when applied to a large cohort of samples in a metabolomics study, comprising of twenty-seven 96-well microplates per nESI-DIMS assay. Specifically, the modification was as follows: after the extraction of each pair of 96-well microplates containing polar metabolites, these two microplates were dried together for 2 h at 35 °C. The extraction process was repeated for the next pair of 96-well microplates as soon as the first pair of microplates started drying.

The samples used here originated from control data from a high-throughput metabolomics study consisting of 1620 nESI-DIMS infusions collected within ~7 days per assay. The study included three biological culturing replicates at 24 h located across three different 96-well microplates. Each biological replicate consisted of technical replicates, i.e., cells in the wells of the same 96-well microplate (*n* = 9 for control samples). Therefore, this study also captured biological variation originating from splitting the cells during the culturing process, which is an additional potential source of variation compared to the workflow evaluation reported above.

**Figure 3:**
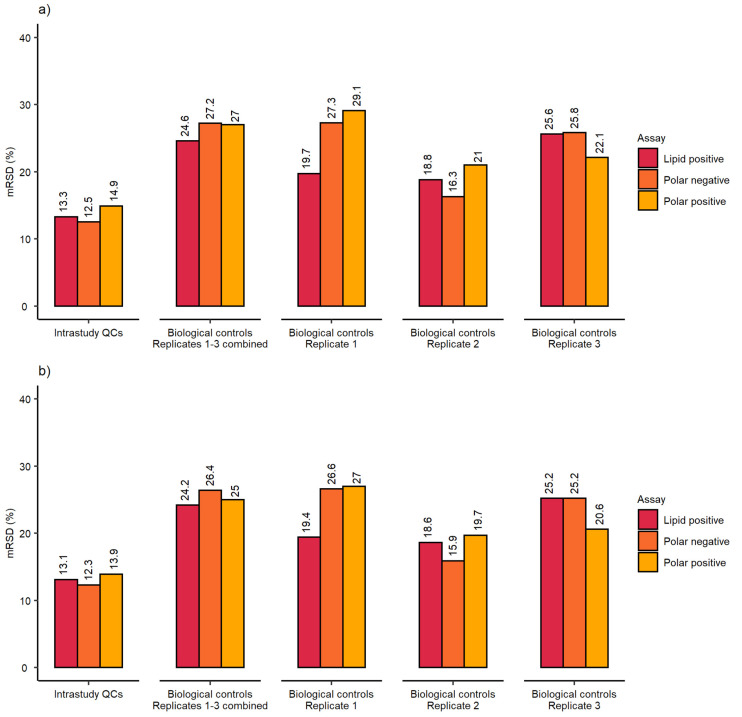
mRSD (%) of spectral feature intensities using control samples at 24 h from the high-throughput metabolomics study (**a**) before and (**b**) after RSD filtering for HepaRG control samples: *n* = 3 biological control replicates (here defined as replicates prepared across multiple weeks of cell culture), each measured as *n* = 9 technical replicates (here defined as replicates wells of cells cultured within the same test 96-well microplate).

The analytical variation of this dataset (derived from analysing ca. 160 intrastudy QC samples across 5 analytical batches) was highly satisfactory with the final mRSD < 15% for datasets after PQN and RSD filtering (Figure 3a,b). The polar positive assays revealed the highest variation, which was slightly lower than reported above—most likely due to small modifications related to liquid handling by the Biomek i7 (e.g., reducing the pipetting speed and height when realiquoting the intrastudy QC samples after preparing the pool). Given the study involved three separate culturing replicates, the total (biological and analytical) variation was also acceptable with the final dataset achieving mRSD < 30% after PQN and RSD filtering across all three nESI-DIMS assays. Additionally, the variation was visualized using principal component analysis (PCA) score plots (Figure S1), which demonstrated a clustering of intrastudy QC samples relative to the biological control samples, across all three nESI-DIMS assays (Figure S1). Biological control samples were relatively tightly clustered for the polar positive assay, yet more dispersed for the two other assays. This was not caused by biological variation originating from splitting the cells during the culturing process, as each of the biological replicates (1–3) showed this pattern.

**Figure 4:**
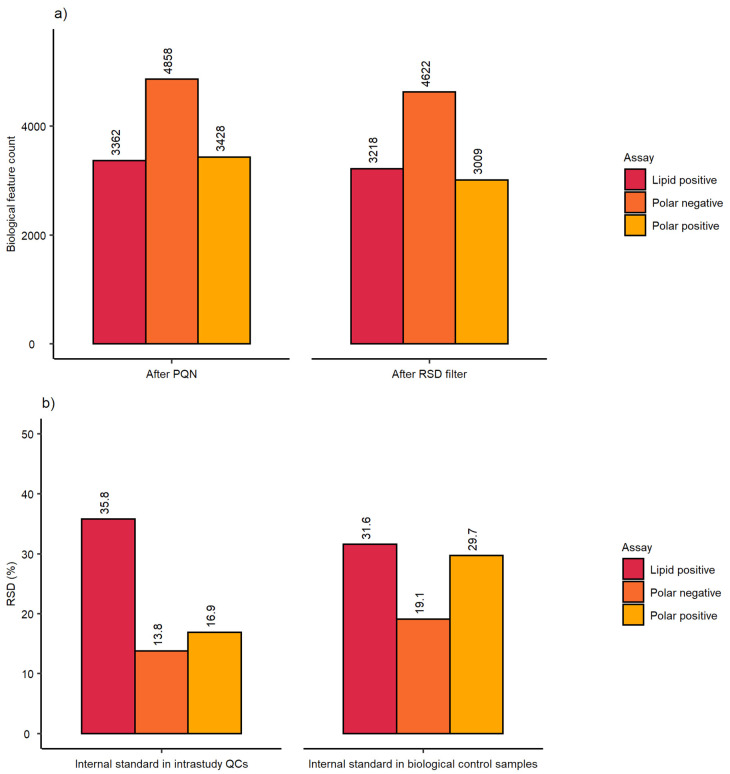
Assessment of (**a**) sensitivity and (**b**) workflow repeatability from the high-throughput metabolomics study by employing biological feature count and the intensity of putatively annotated features of the internal standards across 3 nESI-DIMS assays. Sensitivity and repeatability in intrastudy QC samples were determined on the whole dataset (ca. 1620 samples), whilst repeatability in biological samples employed a subset dataset comprising of control (unexposed) samples at 24 h (bottom right bar chart).

The analytical sensitivity was comparable with small batch studies achieving a biological feature count in the range of 3009–4622 for all three nESI-DIMS assays (Figure 4a). Finally, the RSD (%) of each feature putatively annotated to the internal standards was also calculated (Figure 4b). The RSDs (%) met the threshold criteria for intrastudy QCs in the two polar assays, however the lipid assay revealed much higher variation based on the feature representing dodecylphosphorylcholine-d_38_ standard (35.8%). Similarly, the RSD of this feature in the biological controls at 24 h was also high (31.6%), whilst the polar data demonstrated somewhat lower variation (RSDs of 29.7% and 19.1% for positive and negative ion modes, respectively).

## 3. Discussion

### 3.1. Assessment of Automated Sample Preparation Workflow for Metabolomics

Implementing in vitro metabolomics into chemical risk assessments as NAMs, for example for the purpose of chemical grouping, requires an improvement of the throughput of traditional metabolomics approaches for sample preparation and data acquisition. Simultaneously, the quality of the metabolomics data produced should not be compromised (i.e., no reduction in detection sensitivity and repeatability) to achieve this higher throughput. Here, we propose an automated workflow for in vitro metabolomics that is compatible with 96-well microplates (with one well corresponding to approximately only 50,000 HepaRG cells). The workflow was thoroughly characterised with respect to detection sensitivity and repeatability across three nESI-DIMS assays. Furthermore, the experimental conditions such as the day and order of metabolite extraction from 96-well microplates, well location on the microplate and the position of a 96-well microplate on the sample preparation platform’s deck were assessed by applying statistical analysis. Further adjustments to this workflow were made to improve its quality, which were then demonstrated in a large cohort metabolomics study for three nESI-DIMS assays.

The characterisation of the automated platform for intracellular metabolite extraction and analysis was based upon established metrics: total spectral feature count (after multiple filtering steps conducted to retain only reproducibly detected spectral features of biological origin) and mRSD (%) of spectral features across the whole dataset [4,22]. As shown in previous studies using nESI-DIMS on much smaller sample batches, the total spectral feature count reported in Table 1 was comparable to the literature. For higher biomass samples, e.g., 1 mg of *Daphnia pulex-pulicaria*, Southam et al. [16] reported the detection of 1973 and 2934 spectral features for polar negative and lipid positive assays, respectively. In comparison, the automated in vitro metabolomics workflow described here uses only 50,000 cells per sample. We previously reported a metabolomics workflow for low biomass samples in 96-well microplates that comprises of monophasic extraction of metabolites using low volumes of solvents and removal of the step of splitting a single sample to smaller aliquots before drying for multiple nESI-DIMS assays. Omitting this step allows for drying a larger volume of sample thus improving analytical sensitivity [25]. The potential disadvantage of this approach is the higher number of samples that need to be prepared (i.e., one sample for each nESI-DIMS assay—one sample corresponds to only ca. 50,000 hepatocytes of HepaRG), and therefore it will only be suitable for applications where samples can be cultured at scale. However, in this study, the cell samples were indeed cultured and sampled using a high-throughput automated platform (Hamilton Star and Starlet platforms), streamlining the whole workflow from generating samples to data acquisition, thus making it amenable with other high-content assays used in toxicology. The reported mRSD for intrastudy QC samples are below the threshold of 20% for the final dataset after RSD filtering (17.3%, 7.8% and 12.8% for polar positive, polar negative and lipid positive assays, respectively), which is also comparable with the results reported by Southam et al. (2017) for data collected using an Orbitrap Elite (13.5% for polar serum extract) and Q Exactive (16% for polar human bronchial epithelial cell line extract) mass spectrometers. Similarly, Kirwan et al. [27] also reported similar values for uncorrected mRSD of intrastudy QC samples (~9–17%) for data collected using an LTQ FT Ultra nESI-DIMS. The mRSD of control samples was slightly higher for polar positive (27.6%) and lipid positive (23.6%) assays than reported by Parsons et al. [26] for the K562 cell line (20.5% for untreated cells), however those data were collected using nuclear magnetic resonance (NMR) spectroscopy with lower analytical variation than nESI-DIMS. The mRSD threshold for this study was set to 30% for control samples therefore meeting the commonly accepted criterion [4]. The results shown here for control samples are consistent with the values we previously reported for the same sample type and solvent system: 25.2% and 25.6% for polar positive and lipid positive nESI-DIMS assays respectively [25].

Each 96-well microplate was evaluated on an individual basis revealing acceptable mRSD (%) for tested microplates after applying the RSD filtering. Polar negative assay performed best (achieving lowest mRSD for each microplate: 11.9–16.9% after RSD filtering), however, lipid positive assay produced the most consistent results on a microplate-to-microplate basis with slightly higher mRSDs (22.2–23.8% after RSD filtering).

In this study, isotopically labelled internal standards were used to help determine outlying samples in the datasets by calculating the median and MADs of the features, putatively assigned to protonated or deprotonated analytes within ±5 ppm mass error. The intensities of these features were assessed separately for intrastudy QCs and control samples. In addition, the RSD of this feature was calculated, which followed the trends observed for the mRSD of all spectral features: polar positive assay produced the highest RSD (12.6%), followed by lipid positive (7.1%) and polar negative (5.4%) assays in intrastudy QCs. Similarly, polar positive assay performed most poorly (19.4%) in the spiked control samples followed by polar negative (16.0%) and lipid positive (14.6%) assays as shown in Table 2. Calculating the RSD of this feature helped to assess the performance of the automated workflow without the contribution of variation originating from cell culturing and treatment. However, given this is the assessment of a single feature of one internal standard per assay only, caution should be taken when interpreting the data. In this case, the results reported for this feature supported the observations made when using mRSDs across the whole dataset and in each test microplate.

Statistical analysis (one-way ANOVA followed by post-hoc testing as well as calculations of the fold changes) was used to evaluate if any of the experimental conditions tested resulted in a high percentage of significantly changing features across control samples. The experimental conditions evaluated included the day of metabolite extraction, the order of extraction of samples in 96-well microplates, the position of the 96-well microplate on the instrument’s deck and well location within a microplate (Figure 2 and Table S1). The most prominent differences originated from the extraction order of 96-well microplates for the polar data, indicating that the wait before drying a microplate resulted in significant changes to metabolite intensities post-extraction. Consequently, this step of the workflow was modified when applied to the large cohort study by drying one pair of 96-well microplates as soon as they were prepared. Other conditions such as extraction day and position of a microplate on the sample preparation platform’s deck resulted in a lower percentage of ill-behaved features outside the applied threshold. Finally, it was found that well location (edge vs. centre of a 96-well microplate) did not contribute in a significant manner, thus allowing for the use of all 96 wells in future studies.

### 3.2. Demonstration of the Developed Workflow for High-Throughput Metabolomics Studies

The optimised workflow was applied to a large cohort study (1620 sample infusions per nESI-DIMS assay). Here, we present the control data so that comparisons could be made to results presented earlier after modifications to the initial setup. The results presented in Figure 3 suggest very good repeatability of the whole workflow, which also included three culturing biological replicates prepared by splitting a common cell pool. This step, in theory, could result in a higher variation observed when compared to results presented earlier, which included only one true biological replicate (no split from the common cell pool). The results in Figure 3 met the threshold of 30% for mRSDs both before and after RSD filtering, with the lipid positive method performing best across the three assays applied when three biological replicates were considered together. The analytical precision calculated using intrastudy QCs (measured across multiple days and batches) was also acceptable, achieving mRSDs < 15% for all three assays even before removing more variable features. The analytical sensitivity was comparable to the results presented earlier, achieving final biological feature counts above 3000 even after multiple filtering steps conducted on thousands of samples (Figure 4a).

The repeatability of the internal standard was worse than observed earlier with the lipid positive assay performing most poorly, as shown in Figure 4b (RSDs of 35.8% and 31.6% for intrastudy QCs and all biological control samples at 24 h, respectively). These results suggest that this internal standard (dodecylphosphorylcholine-d_38_) may not be optimal for high-throughput studies with metabolite extractions and analysis performed across multiple days (e.g., due to compound stability). Nonetheless, the internal standard performed well at assisting in the removal of outlying samples, as the final mRSD measured across all biological features was highly satisfactory for the control samples at 24 h as well as all intrastudy QCs. When compared to other high-throughput studies such as the workflow proposed by Smith et al. [14], the approach described here outperforms the methods proposed by the authors with respect to biological feature count and mRSD (%), as the authors reported approximately 2000 spectral features for the control sample set and mRSD of 24.8% for technical replicates. Meister et al. [13] reported similar values to results shown here with mRSD of the intrastudy QCs achieving 10–20% for the largest number of features measured (using LC-MS). The five standards chosen by the authors performed much better than the internal standards used in this study when considering their large cohort scale study. However, some internal standards performed worse than the others (e.g., fluorocinnamic acid and tricarballylic acid with RSDs of 20% and 18%, respectively, for the small cohort) highlighting the importance of including multiple internal standards to more thoroughly evaluate the repeatability of the automated workflow.

## 4. Materials and Methods

### 4.1. Assessment of Automated Sample Preparation Workflow for Metabolomics

#### 4.1.1. Cell Culture and Treatment

Undifferentiated HepaRG cells (HPR101, Biopredic International, Rennes, France, batch HPR-101056) were cultured in 96-well microplates as described previously [28]. Hepatocytes (5 × 10^4^ cells/well in 100 μL medium) were incubated in 0.1% dimethyl sulfoxide (DMSO) (*v*/*v*) corresponding to (negative) control samples as used in toxicological studies. After 24 h, the cell media were discarded followed by washing the adherent cells twice with 180 µL 0.9% ice-cold sodium chloride (*w*/*v*; Fresenius Kabi, Isola della Scala, Italy) and once with 200 µL ice-cold water (sterile-filtered, BioReagent, Sigma, Darmstadt, Germany) using ELx405 microplate washers (BioTek Instruments, Winooski, VT, USA). Water washes were conducted rapidly (<20 s) as suggested by Deng et al. [29]. Next, microplates were sealed with foil (Biorad, Hercules, CA, USA) using an X-Seal Manual Variable Temperature Thermal Sealer at 180 °C for 5 s, placed on dry ice until frozen and stored at −80 °C. The extraction blanks were generated in the same manner, except they did not contain hepatocytes in the wells, only cell media. Cell seeding, serial dilution of chemicals and cell treatment were fully automated and were performed with Hamilton Star and Starlet robotic platforms (Hamilton Italia Srl, Agrate, Brianza, Italy). These liquid handlers were contained in a laminar flow hood and equipped with 96-multichannel heads.

#### 4.1.2. Automated Metabolite Extraction

**Figure 5:**
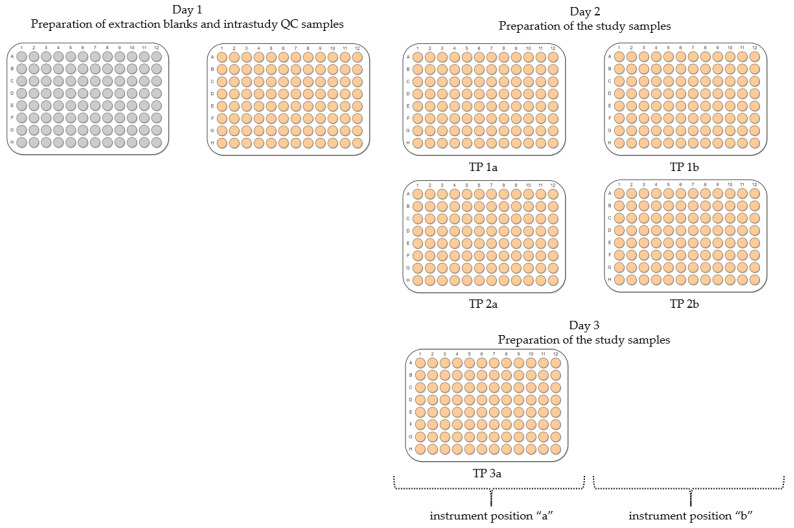
An overview of the experimental design for assessing the automated sample preparation workflow for in vitro metabolomics. Extraction blanks and intrastudy QC samples were prepared on day 1 of the experiment, whilst biological control samples were prepared on days 2 and 3. Conditions tested included the location of a 96-well microplate (containing frozen cells for extraction) on the instrument’s deck (indicated as “a” or “b”), day and order of metabolite extraction as well as the location of samples within a microplate (edge vs. centre).

The experimental design included an assessment of the developed workflow with respect to its repeatability, as well as the statistical analysis of the following experimental conditions: the day of sample preparation, the location of a microplate with samples on the instrument’s deck (denoted as “a” or “b”) and the location of a sample within a microplate (edge or centre) as shown in Figure 5. The order of preparation of samples in microplates was only tested for polar metabolites. The method described below builds on our previously published work [25]. The extraction of metabolites was conducted using an automated laboratory workstation, Biomek i7 (Beckman Coulter, Brea, CA, USA). The Biomek i7 was customised for in vitro metabolomics experiments and equipped with one 96-multichannel head, one Span-8 pod and two grippers. The deck included six tip loading positions, eight automated labware positioners (ALPs) pre-cooled to either −15 °C or 4 °C connected to a Thermo Scientific ARCTIC A40 refrigerated circulator, three Peltier modules pre-cooled to 4 °C for solvent reservoirs, two orbital shakers, sixteen ALPs at room temperature, one wash station and one trash container for used pipetting tips. The Biomek i7 was fully enclosed in a custom-built ventilated cabinet, allowing working with biological samples and volatile solvents (Bigneat, Waterlooville, UK). Custom-made uncoated-aluminium solvent reservoirs ensured minimal leaching of plasticisers. The design and photo of the Biomek i7 deck are shown in Figures S2 and S3.

Polar metabolites were extracted using 4:1 (*v*/*v*) pre-made methanol:water spiked with 1.5 µM L-tryptophan-d_5_ solution (Sigma). Lipids were extracted using 2:1 (*v*/*v*) methanol:chloroform where methanol was spiked with 1.25 µM dodecylphosphorylcholine-d_38_ solution (Sigma). LC-MS grade methanol and water were obtained from Honeywell (Charlotte, NC, USA) and Merck (Darmstadt, Germany), respectively. HPLC-grade chloroform (≥99.8% stabilised with 2-methyl-2-butene) was purchased from VWR Chemicals. The extraction solvents were pre-cooled in the fridge and decanted into uncoated aluminium solvent reservoirs located on Peltier modules at 4 °C. Two 96-well microplates were extracted in parallel, starting with the placement of the first microplate with washed and frozen cells on the ALP precooled to −15 °C, which was followed by the automated metabolite extraction. For polar metabolites, 60 µL of 4:1 (*v*/*v*) methanol:water containing the internal standard was added to each well of the microplate and 30 µL of that aliquot was pipetted up and down five times to promote cell lysis and mixing of the well contents. Forty µL aliquots were transferred to a polypropylene “collection” microplate (Eppendorf, Hamburg Germany) which was followed by the addition of 60 µL of extraction solvent to the original microplate, for a second extraction of the cells. The same volume was then transferred to the “collection” microplate. The steps were repeated for the second 96-well microplate. Both “collection” microplates containing metabolite extracts were moved by grippers to orbital shakers (200 rpm, room temperature, 2 min), then the microplates were moved by an operator to a centrifuge (3622× *g*, 3 min, 4 °C, Sigma 6–16KL). The centrifuged samples were placed on ALPs at room temperature and 80 µL of supernatant per well was transferred to the final 96-well microplate for drying using a centrifugal concentrator (SPD111V230, Thermo Scientific Savant, Waltham, WA, USA) at 35 °C with a 6-microplate rotor. For the evaluation of the automated workflow for polar metabolites, the first pair of extracted microplates waited 40 min for drying to investigate any effects of this delay on metabolite intensities between pairs of microplates extracted as first or last.

For lipids, 60 µL of methanol with spiked internal standard was added to each well of a microplate and the contents of the wells (30 µL) were pipetted up and down five times to promote cell lysis and mixing of the well contents. Forty µL aliquots were then transferred to a “collection microplate”. Next, 40 µL of spiked methanol was added again to the original microplate (second extraction of cells) and the same volume was transferred to the “collection” microplate which was followed by the addition of 40 µL of chloroform. The next steps were conducted as described for the polar metabolites, except the supernatant volume was 96 µL for lipid samples, which were then dried using nitrogen blowdown (Techne Dri-Block DB100/3 sample concentrator) one microplate at a time at 35 °C.

For high-throughput metabolomics, intrastudy QC samples and process extraction blanks were prepared on day 1 of the experiment. Intrastudy QC samples were prepared by extracting metabolites from representative 96-well microplates cultured in the same manner as the biological study microplates. The extraction workflow was also the same as for the study microplates with the exception of pooling individual wells of supernatant into a polypropylene reservoir (Beckman Coulter) at 4 °C, mixing that intrastudy QC pool multiple times, and then realiquoting the pool into aliquots of 80 µL (polar) or 96 µL (lipids) per well and taking the microplates for drying. The process extraction blanks were prepared in the same manner as the intrastudy QC samples.

#### 4.1.3. Data Acquisition

All 96-well microplates were resuspended on the same day for the characterisation of the proposed workflow. The study consisted of eighteen replicates per microplate with nine samples located in the centre of a microplate, and nine samples located at the edges. The dried metabolite extracts were resuspended in 30 µL of 4:1 (*v*/*v*) methanol:water with 0.25% (*v*/*v*) formic acid (~98%, Honeywell) or 4:1 (*v*/*v*) methanol:25 mM aqueous ammonium acetate (≥99.99% trace metals basis, Honeywell) for the polar nESI-DIMS assay in positive and negative ionisation modes, respectively. The lipid extracts were resuspended in 40 µL of 2:1 (*v*/*v*) 7.5 mM methanolic ammonium acetate:chloroform for analyses in positive ionisation mode. The resuspended extracts were centrifuged (3622× *g*, 3 min, 4 °C; Sigma 6–16KL) and the supernatants (20 µL) were then transferred to a 384-well microplate. The step was followed by another centrifugation prior to the nESI-DIMS analysis (2000× *g*, 10 min, 4 °C; Sigma 6–16KL).

The data were acquired using spectral-stitching nESI-DIMS with an Orbitrap Elite mass spectrometer (Thermo Scientific) coupled to a chip-based nESI ionisation platform (TriVersa NanoMate, Advion, Ithaca, NY, USA). The spectral-stitching nESI-DIMS method was developed by Southam et al. [16,17] and modified by Malinowska et al. [25] for low biomass samples. The approach was further optimised for high-throughput metabolomics analyses by decreasing the number of microscans from ten to seven, and ten to three, for the analysis of polar metabolites and lipids, respectively, whilst the number of internal replicates was reduced from four to three. Each infusion took ca. 4.5 min, which also includes the time required for the TriVersa NanoMate to aspirate the sample and later dispose of the tip after data acquisition is complete. The details of data acquisition for each assay are included in the Supplementary Materials (Table S2). The intrastudy QC samples were infused after every eight study samples. The order of analysis of the study samples was fully randomised.

#### 4.1.4. Data Processing and Analysis

The DIMS data were processed using DIMSpy tools [30] within the Galaxy workflow management system as described previously by Southam et al. [16] and further modified by Malinowska et al. [25] for low biomass samples by using internal scan replication. Due to the use of three internal scan replicates (instead of four, as described previously), the minimum number of scans for each *m*/*z* window was set to 2, whilst a minimum fraction of scans a peak had to be present in was set to 0.6. After aligning features across the samples, the peak intensity matrix was searched for putative annotations ([M+H]^+^ or [M−H]^−^) of the internal standards within ±5 ppm. The intensity of the selected feature was used to calculate thresholds (separately for intrastudy QC samples and study samples) to identify outlying samples in the dataset (e.g., due to a failed infusion or pipetting error). The separate assessment of intrastudy QCs and study samples was due to slight differences in their preparation. Intrastudy QCs were prepared on day 1 of metabolite extraction using representative 96-well microplates cultured for this purpose. The internal standard was added to the extraction solvent and following steps of the “regular” extraction procedure, the representative samples for making the intrastudy QCs were pooled and realiquoted before drying. Therefore, this sample type captured variation arising from (a) realiquoting the pool, (b) drying down the samples, (c) resuspending these samples and (d) sample analysis. Study samples (biological control samples for this study) were prepared after day 1. The internal standard was added to the extraction solvent, but these samples were not pooled. Therefore, they captured variation starting with the addition of extraction solvent up to the sample analysis. Consequently, the intrastudy QCs and study samples captured different degrees of variation, thus the thresholds discussed next were calculated separately for intrastudy QCs and study samples.

The thresholds were calculated in R studio using putative annotations of adducts of the internal standards as shown below.

(1) $$min/max\;threshold\;\left(QC\right)=median_{ISintensity\;}-/+\left(3\times \;median\;absolute\;deviation_{ISintensity}\right)$$

(2) $$min/max\;threshold\;\left(study\;samples\right)=median_{ISintensity\;}-/+\left(2\times \;median\;absolute\;deviation_{ISintensity}\right)$$

The value of MAD was calculated including the scaling factor of 1.4826 (corresponding to one standard deviation). If the feature was not recorded in a sample and the corresponding intensity was recorded as 0, the calculations of median and median absolute deviations took that into consideration given that the internal standard was expected to be detected in every sample. After removing samples identified by the internal standard as outliers, the workflow was rerun followed by a blank subtraction and feature filtering (i.e., retaining spectral features present in at least 80% of all study samples and intrastudy QCs). The features annotated as adducts and isotopes of internal standards were removed before further data processing and analysis, which consisted of the following steps conducted using StructToolbox [31]. First, samples with a high percentage of missing values were removed (threshold 30%), followed by the removal of features not present in at least 70% of intrastudy QC samples, then PQN was applied and spectral features for which RSDs exceeded 30% in intrastudy QCs were also removed. For multivariate analysis, missing values were imputed using the k-nearest neighbour algorithm (k = 5) followed by generalised log transformation and mean centring. Principal component analysis (PCA) helped to identify additional outliers, which were removed using a 95% confidence interval, and the processing was rerun to generate the final dataset for statistical analysis.

The experiment was designed to evaluate the variability of the workflow with respect to mRSD (%) of each microplate, as well as to investigate if any of the tested experimental conditions resulted in features changing significantly with absolute fold changes above 1.2. The statistical analysis was conducted using StructToolbox [31], which employed one-way ANOVA (*p*-value ≤ 0.05) with FDR using the Benjamini-Hochberg procedure to compare the test 96-well microplates [32]. This was followed by the Tukey-Kramer method for post-hoc analysis as used for unbalanced designs. Fold changes were calculated using geometric means. All analyses were conducted on features detected in at least three replicates per studied group for a condition being evaluated. The results of one-way ANOVA and fold changes were combined for each experimental group and the results were filtered based upon adjusted *p*-values from one-way ANOVA ≤ 0.05. Then, only those features that in post-hoc testing yielded *p*-values of ≤0.05 and absolute fold changes above 1.2 were considered to be outside the set threshold.

Finally, the datasets from this study were putatively annotated using accurate mass measurements by the Python package BEAMSpy (Birmingham mEtabolite Annotation for Mass Spectrometry, https://github.com/computational-metabolomics/beamspy, accessed on 27 May 2021, version 1.1.0). The database employed for annotation of the polar dataset (both ionisation modes) was an in-house HMDB-based list of metabolites prepared by Sostare et al. [33] where exogenous compounds were removed to decrease the rate of false-positive annotations. The database used for the annotation of lipids was LIPID MAPS. The mass error was set to 5 ppm, and the adducts were: [M+H]^+^, [M+Na]^+^, [M+NH_4_]^+^ for positive ionisation mode, and [M−H]^−^, [M+Cl]^−^, [M+Hac−H]^−^ for negative ionisation mode, and the annotated spectral features are included in the Supplementary Materials (Table S3).

### 4.2. Demonstration of the Developed Workflow for High-Throughput Metabolomics Studies

#### 4.2.1. Cell Culture and Treatment

Undifferentiated HepaRG cells (HPR101, Biopredic International, Rennes, France, batch HPR-10101067) were cultured as described above (Section 4.1.1). The study incorporated three biological and nine technical culturing replicates for control samples (27 replicates in total). Definitions differ to what is commonly meant by these terms in metabolomics studies; for this study, biological replicates incorporated cells split during the study and subsequently cultured on three separate weeks using separate 96-well microplates, whilst technical replicates comprised samples on the same 96-well microplate (see Figure S4). Untreated cells (*n* = 9) per 96-well microplate were incubated in 0.1% DMSO. Intrastudy QC samples and extraction blanks were composed of three biological replicates. These samples were on separate 96-well microplates to the study samples (Figure S5).

#### 4.2.2. Automated Metabolite Extraction

The metabolite extraction was conducted as described in Section 4.1.2, with the exception of the process of drying polar metabolites. Each pair of microplates containing polar extracts was dried for 2 h immediately after the supernatant was transferred to the “collection” 96-well microplates. Small modifications aiming to improve the precision of liquid handling were also conducted (e.g., speed and height of pipetting). The extraction blanks and intrastudy QC samples were prepared on day 1 by pooling the supernatant of representative samples allocated for this purpose; the pool was then realiquoted as shown in Figure S5 and dried. The study samples in 96-well microplates were extracted on days 2–4; the order of the 96-well microplates was randomised and the workflow for this study is shown in Figure S6.

#### 4.2.3. Data Acquisition

On the day of mass spectrometric analysis, one dried 96-well microplate with intrastudy QC samples and extraction blanks and three study 96-well microplates were resuspended, centrifuged and transferred to a 384-well microplate for nESI-DIMS analysis, as described above (Figure S6). The order of resuspension of 96-well microplates and sample analysis was randomised. A mixture of metabolite standards of known composition and concentration was analysed at the beginning and end of each sequence to assess potential drift of mass accuracy by the mass spectrometer.

#### 4.2.4. Data Processing and Analysis

The data processing and analysis were similar to the methods described in Section 4.1.4. Given the high-throughput nature of the study, a mixture of metabolite standards and the internal standard in every sample (with the exception of extraction blanks) was used to analyse for any drift of the mass spectrometer with respect to mass accuracy. Indeed, a drift was observed across all three nESI-DIMS assays, requiring modification of the existing data processing workflow. This modification was implemented after the first step in the processing workflow (i.e., “process scans”) using 1-D smoothing spline fit and a leave-one-out cross-validation. The correction was conducted using *m*/*z* data corresponding to the [M+H]^+^ or [M−H]^−^ of the internal standards. To ensure that the drift was observed across the full range of measured *m*/*z* values (and not just a single spectral feature), features arising from metabolites expected to be present in the premade mixture of metabolite standards were searched for using putative annotations and compared against the trends observed for the features putatively annotated as the internal standards. After the alignment of features, the putative annotations of the internal standards (one per nESI-DIMS assay) were used to remove outlying samples where the internal standard intensity was more than 3× MAD away from the median.

(3) $$\min/\max\;\mathrm{threshold}={\mathrm{median}}_{\mathrm{ISintensity}\;}-/+\left(3\times {\;\mathrm{median}\;\mathrm{absolute}\;\mathrm{deviation}}_{\mathrm{ISintensity}}\right)$$

The processing workflow was then rerun without the outlying samples. Following the removal of features of non-biological origin, only features measured in at least 50% of all samples were retained. Next, samples with the percentage of missing values exceeding 40% for the polar positive assay and 50% for the polar negative and lipid positive assays were removed. This step was followed by retaining the features present in at least 70% of intrastudy QC samples and correcting any drift in signal intensities using the StructToolbox R package [31]. The corrected dataset was then normalised using the PQN method and features with RSDs exceeding 30% (i.e., high technical variability) were removed [34]. Initial data evaluation suggested that the 96-well microplate corresponding to biological replicate one at 48 h was outlying compared to all other samples (observed across all three nESI-DIMS assays). For this reason (and supported by concerns that this microplate was located at the top of the package of microplates shipped from Italy to the UK on dry ice), these samples were removed and the workflow rerun without the outlying 96-well microplate. For the purpose of this study, only the data corresponding to the untreated cells at 24 h were used (including biological and technical culturing replicates). The evaluation of the quality of this dataset was based upon mRSD (%) of all features in control samples at 24 h as well as intrastudy QC samples across the whole dataset. In addition, total spectral feature count and RSD (%) of the feature putatively annotated as the internal standard—separately for polar metabolites and lipids—were reported. For the preparation of PCA plots to evaluate trends in the data, missing values were imputed using the k-nearest neighbour algorithm (k = 5) followed by the generalised log transformation and mean centring.

## 5. Conclusions

We have developed, characterised and demonstrated an automated sample preparation and analysis workflow for in vitro metabolomics in 96-well microplates (with each sample corresponding to only 50,000 hepatocytes of HepaRG), using a Biomek i7 Hybrid Workstation (Beckman Coulter) and spectral-stitching nESI-DIMS (Thermo Scientific Orbitrap Elite) for polar metabolites and lipids. The biological feature count, and analytical and biological variation, were highly acceptable, achieving our target criteria for the majority of studied parameters and conditions, but some modifications to the proposed workflow were required to achieve optimum performance. The optimised workflow was then applied to a large cohort metabolomics study, again meeting the threshold criteria and demonstrating that this workflow is ready for application. This workflow offers the possibility of increasing throughput of sample preparation, in principle allowing for metabolite extraction of up to ca. 18 (polar) or 14 (lipids) study 96-well microplates, assuming an 8-h shift with a single operator. In addition, nESI-DIMS methods for low biomass samples are also well suited to high-throughput analyses allowing the analysis of up to ca. 328 (polar) or 342 (lipid) samples within 24 h using a single instrument. The approach proposed provides a compromise between high data quality (such as total feature count and repeatability) and throughput when compared to other available methods. However, future studies should consider the use of multiple internal standards to further assess the variability of the workflow for metabolites and lipids across the measured *m*/*z* range. It is envisioned that for challenging sample sizes with low biomass, which require rapid analyses, the workflow presented here provides a solution for applying metabolomics to a range of scenarios in chemical risk assessment, particularly high-throughput screening.

## Figures and Tables

**Table 1:** Assessment of analytical sensitivity as well as analytical and biological repeatability of the automated in vitro metabolomics workflow across three nESI-DIMS assays. P(+), P(−), L(+) correspond to polar positive, polar negative and lipid positive nESI-DIMS assays. For the measurement of repeatability, the number of replicates is given in brackets below the mRSD value.

		Dataset after PQN			Dataset after RSD Filter		
Assessment	Parameter	P(+)	P(−)	L(+)	P(+)	P(−)	L(+)
Analytical sensitivity	Spectral feature count	3120	4862	3937	2329	4782	3788
Analytical repeatability	mRSD (%) intrastudy QCs	20.9 (*n* = 14)	7.8 (*n* = 14)	13.1 (*n* = 9)	17.3 (*n* = 14)	7.8 (*n* = 14)	12.8 (*n* = 9)
Biological and analytical repeatability	mRSD (%) biological control samples	31.3 (*n* = 75)	19.5 (*n* = 75)	24 (*n* = 47)	27.6 (*n* = 75)	19.3 (*n* = 75)	23.6 (*n* = 47)

**Table 2:** Assessment of the automated in vitro metabolomics workflow’s repeatability based on a spectral feature putatively assigned to the internal standards: L-tryptophan-d_5_ for polar metabolomics assays and dodecylphosphorylcholine-d_38_ for the lipid assay ([M+H]^+^ or [M−H]^−^ for positive and negative ion modes, respectively). P(+), P(−), L(+) correspond to polar positive, polar negative and lipid positive nESI-DIMS assays. For the measurement of repeatability, the number of replicates is given in brackets below the RSD value.

Assessment	Parameter	Class	P(+)	P(−)	L(+)
Workflow repeatability (excluding cell culture)	RSD (%) of internal standard	Intrastudy QCs	12.6 (*n* = 14)	5.4 (*n* = 14)	7.1 (*n* = 9)
Workflow repeatability (excluding cell culture)	RSD (%) of internal standard	Control samples	19.4 (*n* = 75)	16.0 (*n* = 75)	14.6 (*n* = 47)

## Data Availability

The data presented in this study are available on request from the corresponding authors. The data are not publicly available due to privacy.

## Supplementary Materials

The following are available online at www.mdpi.com/article/10.3390/metabo12010052/s1, Figure S1: PCA score plots of the intrastudy QCs (from 5 analytical batches, collected over ~7 days), and biological control samples, Figure S2: The design of the deck of Biomek i7 customised for in vitro metabolomics experiments, Figure S3: The photo of the deck of Biomek i7 customised for in vitro metabolomics experiments with assigned parts used for in vitro metabolomics workflow, Figure S4: Experimental design for the high-throughput metabolomics study shown for one nESI-DIMS assay, Figure S5: The experimental design outlying the generation of extraction blanks and intrastudy QC samples for the high-throughput metabolomics study, Figure S6: High-throughput metabolomics workflow providing an overview of the process of sample extraction, resuspension and analysis for nESI-DIMS assays using 96-well (for culturing and extractions) and 384-well (for nanoelectrospray into the mass spectrometer) microplates, Table S1: Assessment of the effect of well location during the culturing and extraction procedure, Table S2: Settings of data acquisition employed for the assessment of automated sample preparation workflow for in vitro metabolomics acquired using spectral-stitching nESI-DIMS, Table S3: Lists of putatively annotated spectral features from the study evaluating the sensitivity and repeatability of the automated platform for intracellular metabolite extraction and analysis.
