# Supplementary material for: Automated Sample Preparation and Data Collection Workflow for High-Throughput In Vitro Metabolomics
Source: Metabolites. 2022 Jan 8;12(1):52. doi: 10.3390/metabo12010052 (PMC8778710; doi:10.3390/metabo12010052)
Supplement: Supplementary file 1 [file metabolites-12-00052-s001.zip › 2_SI_AutomatedSamplePrepforMet-proofs-JMM.pdf]

Supplementary material for

Automated Sample Preparation and Data Collection Workflow for High-Throughput In Vitro Metabolomics

Julia M. Malinowska [1,*], Taina Palosaari [2], Jukka Sund [2], Donatella Carpi [2], Gavin R. Lloyd [3], Ralf J.M. Weber [1,3], Maurice Whelan [2] and Mark R. Viant [1,3,*]

1 School of Biosciences, University of Birmingham, Birmingham B15 2TT, UK; R.J.Weber@bham.ac.uk (R.J.M.W.)

2 European Commission, Joint Research Centre (JRC), 21027 Ispra, Italy; Taina.PALOSAARI@ec.europa.eu (T.P.); Jukka.SUND@ec.europa.eu (J.S.); Donatella.CARPI@ec.europa.eu (D.C.); Maurice.WHELAN@ec.europa.eu (M.W.)

3 Phenome Centre Birmingham, University of Birmingham, Birmingham B15 2TT, UK; G.R.Lloyd@bham.ac.uk (G.R.L.)

* Correspondence: jmm732@bham.ac.uk (J.M.M.); m.viant@bham.ac.uk (M.R.V.)

Contents

Supplementary results 3

Table S1. Assessment of the effect of well location during the culturing and extraction procedure (wells located at the edges or centre of a 96-well microplate) 3

Figure S1. PCA score plots of the intrastudy QCs (from 5 analytical batches, collected over ~7 days), and biological control samples (the latter corresponding to a 24-h sampling time point) 4

Supplementary methods 5

Table S2. Settings of data acquisition employed for the assessment of automated sample preparation workflow for metabolomics 5

Figure S2. The design of the deck of Biomek i7 Hybrid Workstation customised for in vitro metabolomics experiments 7

Figure S3. The photo of the deck of Biomek i7 Hybrid Workstation customised for in vitro metabolomics experiments 7

Figure S4. Experimental design for the high-throughput metabolomics study shown for one nESI-DIMS assay 8

Figure S5. The experimental design outlying the generation of extraction blanks and intrastudy QC samples for the high-throughput metabolomics study 9

Figure S6. High-throughput metabolomics workflow providing an overview of the process of sample extraction, resuspension and analysis for nESI-DIMS assays using 96-well (for culturing and extractions) and 384-well (for nESI-DIMS) microplates 10

Supplementary results

Table S1. Assessment of the effect of well location during the culturing and extraction procedure (wells located at the edges or centre of a 96-well microplate) for each study 96-well microplate using Welch's t-test (*p*-value threshold 0.05, correction for FDR applied using the Benjamini-Hochberg procedure) and fold change calculations (absolute value > 1.2) across three nESI-DIMS assays. For each study 96-well microplate, a feature had to be present in at least three samples per group to apply the statistical analysis. P(+), P(-), L(+) correspond to polar positive, polar negative and lipid positive nESI-DIMS assays.

Assay	96-well microplate	Features outside the threshold	Total biological feature count for the study 96-well microplate	Percentage (%) of features outside the threshold
P(+)	TP 1a	0	2328	0.0
	TP 1b	0	2326	0.0
	TP 2a	167	2327	7.2
	TP 2b	13	2258	0.6
	TP 3a	0	2329	0.0
P(-)	TP 1a	0	4768	0.0
	TP 1b	0	4743	0.0
	TP 2a	2	4781	0.0
	TP 2b	0	4776	0.0
	TP 3a	0	4661	0.0
L(+)	TP 1a	0	3787	0.0
	TP 1b	0	3777	0.0
	TP 3a	0	3788	0.0

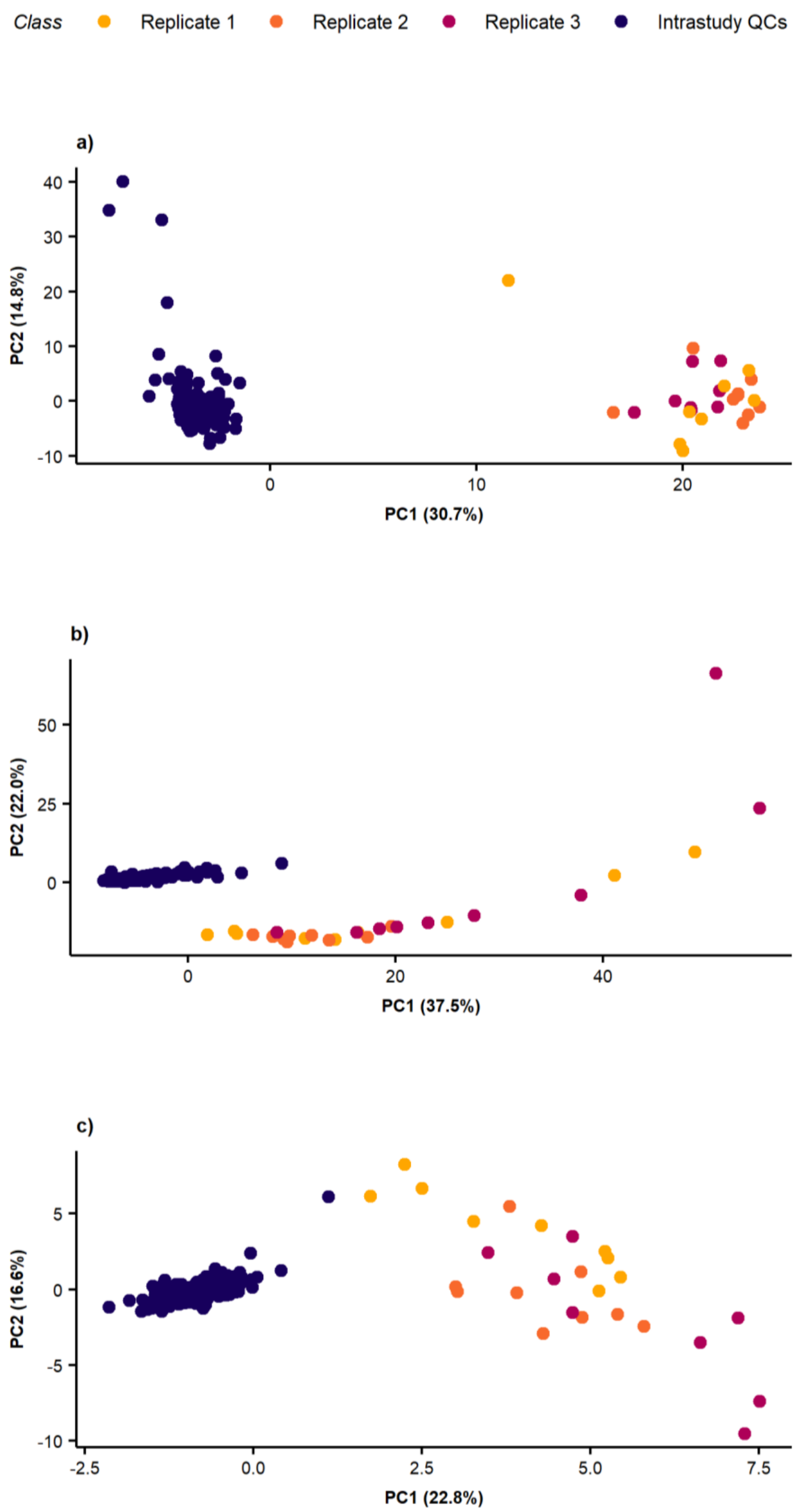

Figure S1. PCA score plots of the intrastudy QCs (from 5 analytical batches, collected over ~7 days), and biological control samples (the latter corresponding to a 24-h sampling time point) for a) polar positive, b) polar negative, and c) lipid positive nESI-DIMS assays from the high-throughput metabolomics study. Biological replicates 1-3 correspond to the cell samples cultured on three separate 96-well microplates across weeks 1-3.

Supplementary methods

Table S2. Settings of data acquisition employed for the assessment of automated sample preparation workflow for in vitro metabolomics acquired with spectral-stitching nESI-DIMS using an Orbitrap Elite mass spectrometer (Thermo Scientific) coupled to a chip-based nESI ionisation platform (TriVersa NanoMate, Advion, Ithaca, USA) for the analysis of polar metabolites in positive and negative ionisation modes as well as lipids in positive ionisation mode.

		Assay		
**Instrument**	**Settings**	**Polar positive**	**Polar negative**	**Lipid positive**
TriVersa NanoMate	Infusion volume (μL)	10	10	10
	Aspirate after sample	Yes	Yes	Yes
	Return unused sample to tray	No	No	No
	Vent headspace	Yes	Yes	Yes
	Volume of air after sample (μL)	0.2	0.3	1.2
	Pressure (psi)	0.30	0.30	0.30
	Voltage (kV)	1.40	1.50	1.20
	Polarity	Positive	Negative	Positive
	Air gap before chip	Yes	Yes	Yes
	Contact closure timing	After sample flow	After sample flow	After sample flow
	Voltage timing	Voltage after	Voltage after	Voltage after
	Equalization delay (s)	0	0	5
	Volume after delivery (μL)	0.5	0.5	0.5
	Aspiration delay (s)	0	0	0
	Contact closure delay (s)	0	0	0
	Voltage delay (s)	0	0	0

	Aspiration depth (mm)	1.2	1.2	1.4
	Use spray sensing	No	No	No
Orbitrap Elite (spectral-stitching)	Stabilisation period for spray (s)	15	15	30
	Number of internal scan replicates	3	3	3
	Number of SIM stitch windows per internal scan replicate	10	10	18
	Maximum ion injection times (ms)	200	200	200
	Peak resolution at *m/z* 400	240,000	240,000	240,000
	Microscans	7	7	3

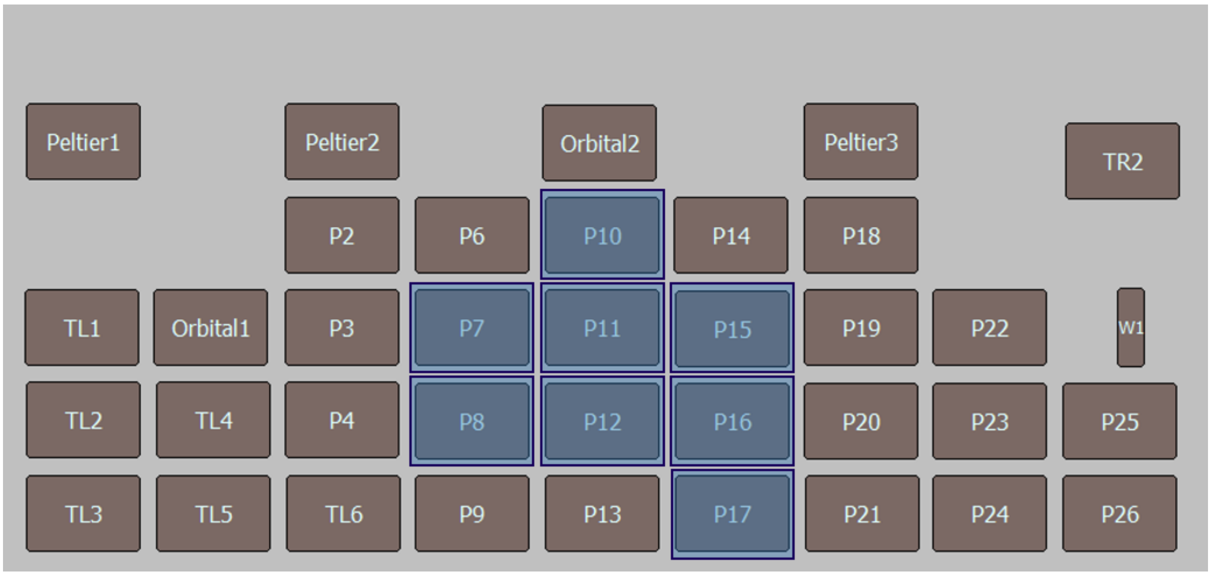

Figure S2. The design of the deck of Biomek i7 Hybrid Workstation customised for in vitro metabolomics experiments. Abbreviations: TL – tip loading stations (1-6), Orbital – orbital shakers (1-2), Peltier – Peltier modules maintained at 4 °C (1-3), W – washing station, TR – trash. Automated labware positioners (ALPs) highlighted in blue were maintained at - 15 °C (metabolite extraction) or 4 °C (metabolite resuspension), the remaining ALPs were maintained at room temperature.

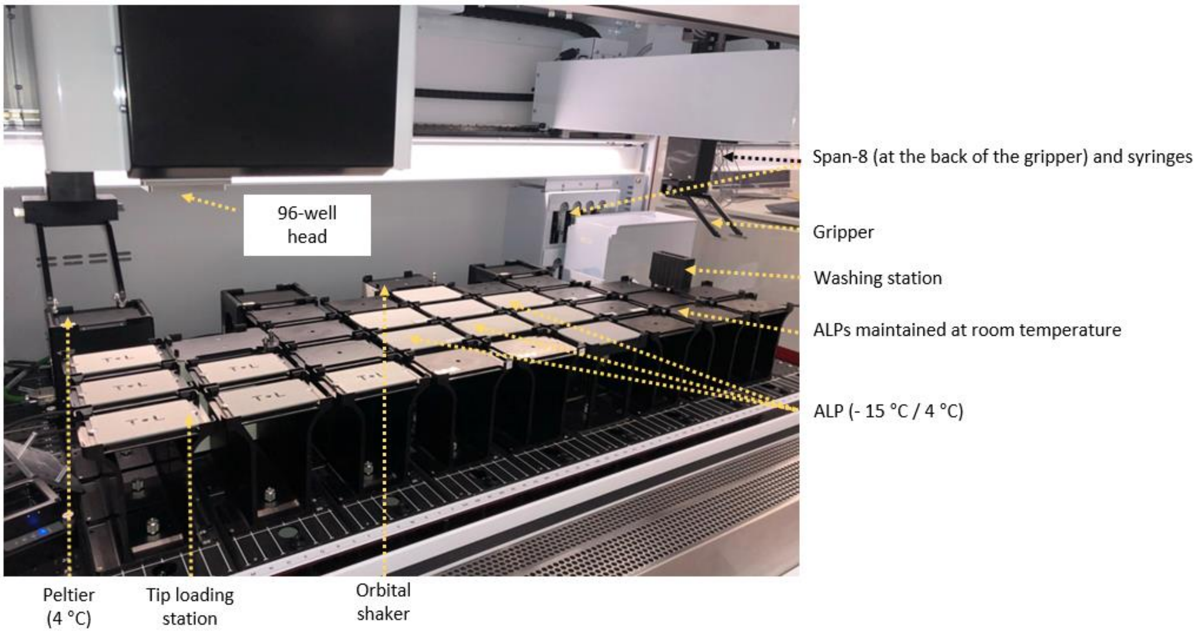

Figure S3. The photo of the deck of Biomek i7 Hybrid Workstation customised for in vitro metabolomics experiments.

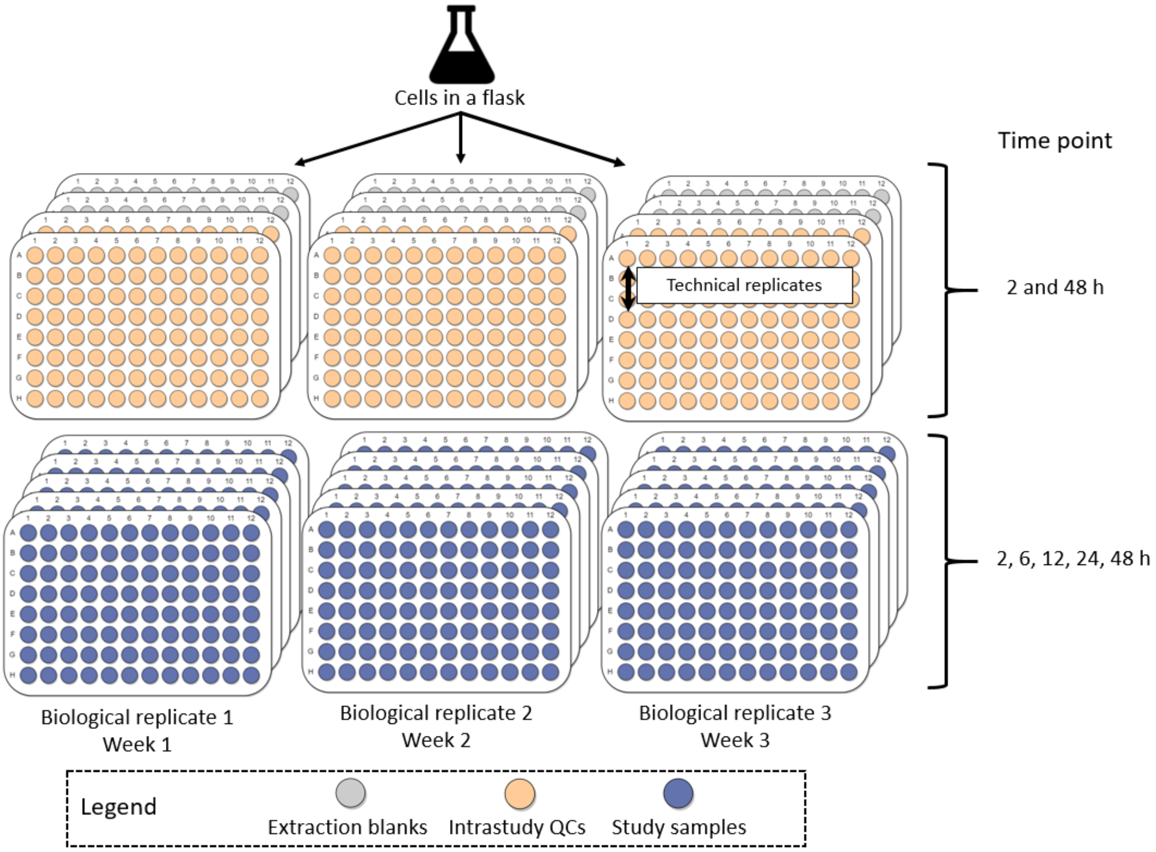

Figure S4. Experimental design for the high-throughput metabolomics study shown for one nESI-DIMS assay. The study included 15 study 96-well microplates with cells exposed to four chemicals across five time points. Untreated control samples (the focus of this paper) were incubated in 0.1% DMSO. The extraction blanks and intrastudy QC samples were prepared in the same manner as for the study samples but only at 2 and 48 h. Biological replicates corresponded to cells split during the culturing process, treated across different weeks (n=3). Technical replicates included cells on the same 96-well microplates (n=9 for untreated control samples).

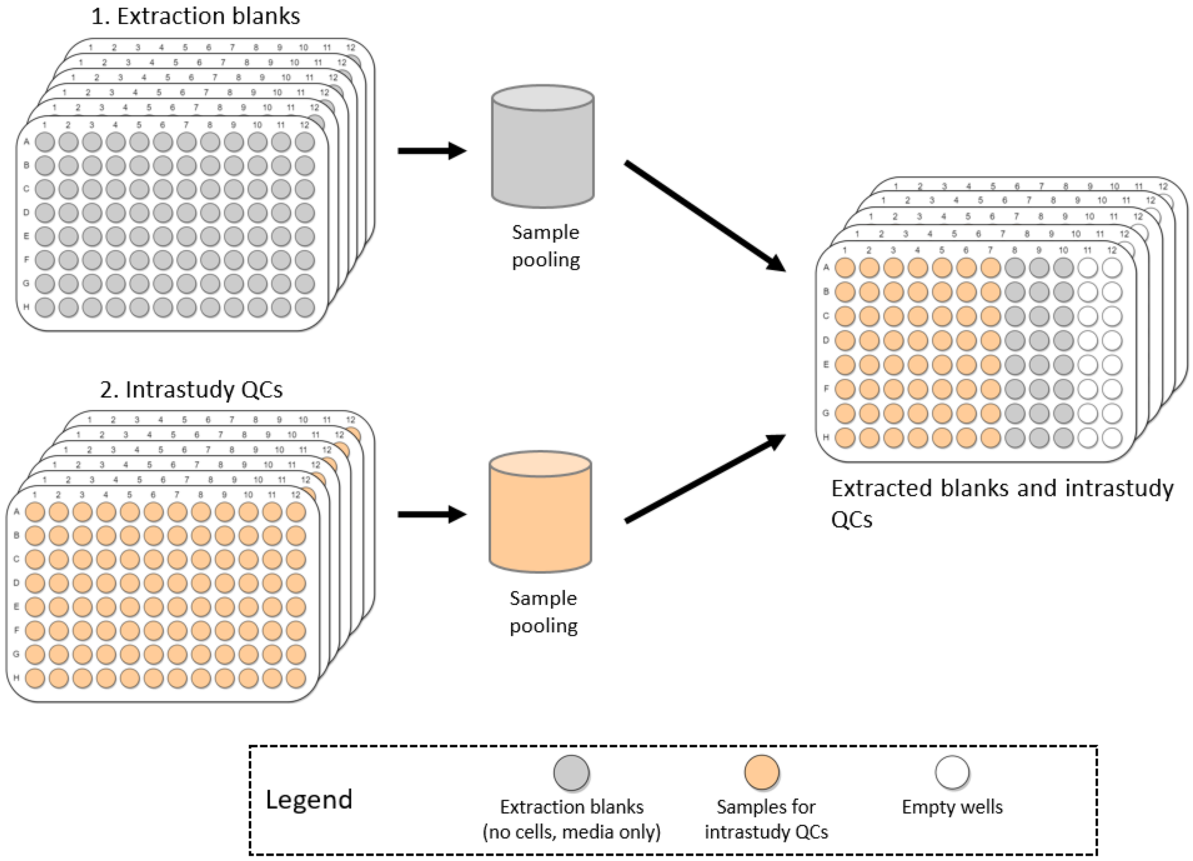

Figure S5. The experimental design outlying the generation of extraction blanks and intrastudy QC samples for the high-throughput metabolomics study. These samples were generated alongside the study samples corresponding to three biological replicates at two time points (2 and 48 h).

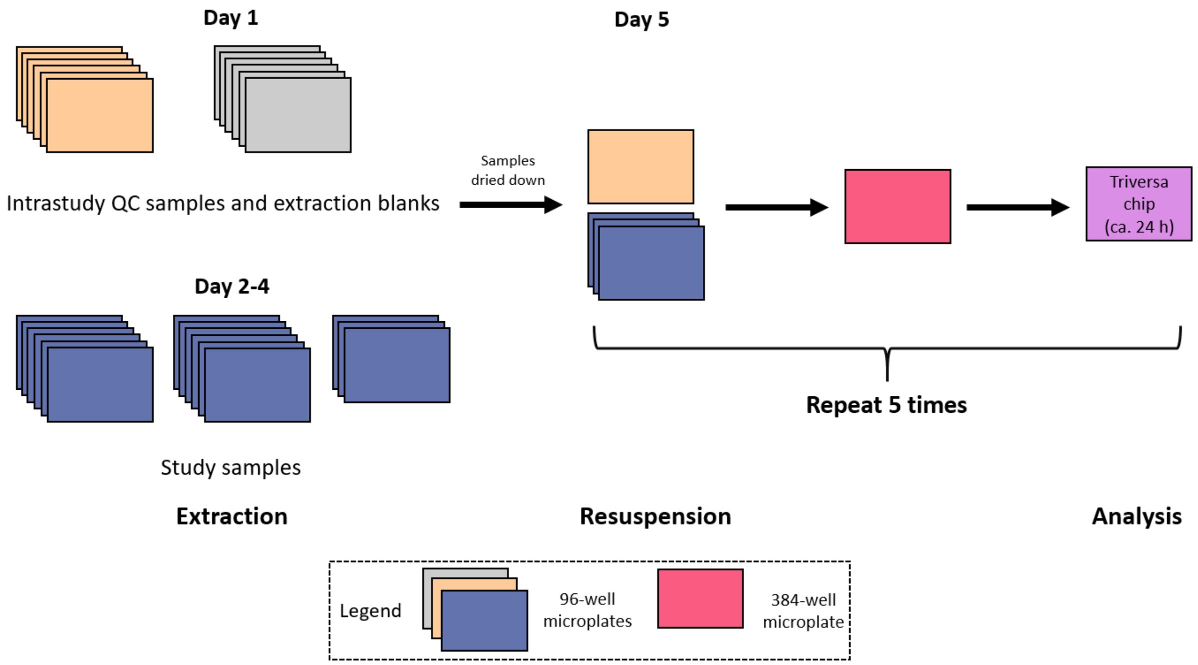

Figure S6. High-throughput metabolomics workflow providing an overview of the process of sample extraction, resuspension and analysis for nESI-DIMS assays using 96-well (for culturing and extractions) and 384-well (for nESI-DIMS) microplates.
